# Complete Genome Sequence of the BeAn 58058 Virus Isolated from *Oryzomys* sp. Rodents in the Amazon Region of Brazil

**DOI:** 10.1128/genomeA.01575-16

**Published:** 2017-03-02

**Authors:** A. L. M. Wanzeller, A. L. P. Souza, R. S. S. Azevedo, E. C. Sousa Júnior, L. C. F. Filho, R. S. Oliveira, P. S. Lemos, J. V. Júnior, P. F. C. Vasconcelos

**Affiliations:** a Department of Virology, Evandro Chagas Institute, Ministry of Health, Ananindeua, Brazil; b Department of Arbovirology and Hemorrhagic Fevers, Evandro Chagas Institute, Ministry of Health, Ananindeua, Brazil; c Center for Technological Innovation, Evandro Chagas Institute, Ministry of Health, Ananindeua, Brazil

## Abstract

Here, we report the complete genome sequence of the BeAn 58058 virus (prototype) strain, isolated from a wild rodent *Oryzomys* sp. in the Utinga forest, Belém, state of Pará, Brazil in 1963. The genome of this virus showed similarity to the *Poxviridae* family, suggesting its inclusion in a possible new genus.

## GENOME ANNOUNCEMENT

Poxviruses are enveloped viruses with mean diameters of 222 to 450 nm long and 140 to 260 nm wide, and the replication occurs in the cellular cytoplasm. The genome is a double-stranded DNA, ranging in size from 130 to 300 kb, and presenting a biconcave core. During viral morphogenesis these viruses presented particles with different stages of maturation. The *Poxviridae* is divided into two subfamilies, *Chordopoxvirinae* (10 genera) and *Entomopoxvirinae* (three genera), that infect vertebrates and invertebrates, respectively, some of which were not classified by genus (1). In recent years, studies have been done to evaluate the genome and, consequently, the reclassification of unclassified poxviruses into a taxon. Presently, few poxviruses have their complete genomes published (2–5).

Recent studies have demonstrated the circulation of strains similar to *Vaccinia virus* infecting and causing disease in many animal groups, including reptiles, birds, and mammals, representing a public health concern worldwide.

The BeAn 58058 virus was originally isolated from a wild rodent *Oryzomys* sp. in the Utinga forest, Belém, state of Pará, Brazil in 1963. The BeAn 58058 virus and others have not been assigned to a genus in the *Poxviridae* family.

Preliminary serological studies have demonstrated that the BeAn 58058 virus presented reactivity against its homologous and Cotia virus sera (6) and molecularly it was classified as a member of the *Orthopoxvirus* genus (6, 7).

This virus strain was grown in Vero cells and processed to TEM (8) to study its genome using the GS FLX 454 (Roche, Life Science) and Ion Torrent platforms, and the entire genome was sequenced. The supernatant of Vero cells was precipitated using polyethylene glycol (PEG) as previously described and the clarified supernatant treated with DNase and RNase (Ambion) for host contaminant debris removal. Treated samples were further used for RNA extraction and for full-length genome sequencing. The genome was obtained employing a *de novo* hybrid assembly strategy using the two platforms mentioned above.

By negative staining and ultrathin section we observed virus enveloped particles similar to poxviruses in the cytoplasm with different stages of maturation. The total genome recovered was 163,005 nucleotides (nt) in length with a mean coverage of 79× fold.

This is the first report of the complete genome sequence for the BeAn 58058 virus strain isolated in northern Brazil. BeAn 58058 was separated into a clade with the Cotia virus, another member of the *Poxviridae* family, thus, we propose that these two viruses constitute a new genus in this family called the *Cotiapoxvirus*.

### Accession number(s).

The complete genome has been deposited in GenBank under the accession no. KY094066.
